# CRISPR-resolved virus-host interactions in a municipal landfill include non-specific viruses, hyper-targeted viral populations, and interviral conflicts

**DOI:** 10.1038/s41598-023-32078-6

**Published:** 2023-04-05

**Authors:** Nikhil A. George, Laura A. Hug

**Affiliations:** Department of Biology, University of Waterloo, Waterloo, ON Canada

**Keywords:** Metagenomics, Virus-host interactions, Environmental microbiology

## Abstract

Viruses are the most abundant microbial guild on the planet, impacting microbial community structure and ecosystem services. Viruses are specifically understudied in engineered environments, including examinations of their host interactions. We examined host-virus interactions via host CRISPR spacer to viral protospacer mapping in a municipal landfill across two years. Viruses comprised ~ 4% of both the unassembled reads and assembled basepairs. A total of 458 unique virus-host connections captured hyper-targeted viral populations and host CRISPR array adaptation over time. Four viruses were predicted to infect across multiple phyla, suggesting that some viruses are far less host-specific than is currently understood. We detected 161 viral elements that encode CRISPR arrays, including one with 187 spacers, the longest virally-encoded CRISPR array described to date. Virally-encoded CRISPR arrays targeted other viral elements in interviral conflicts. CRISPR-encoding proviruses integrated into host chromosomes were latent examples of CRISPR-immunity-based superinfection exclusion. The bulk of the observed virus-host interactions fit the one-virus-one-host paradigm, but with limited geographic specificity. Our networks highlight rare and previously undescribed complex interactions influencing the ecology of this dynamic engineered system. Our observations indicate landfills, as heterogeneous contaminated sites with unique selective pressures, are key locations for atypical virus-host dynamics.

## Introduction

Viruses are important and understudied members of microbial ecosystems that, with the advent of direct community shotgun sequencing, can now be studied at much higher throughput. Metagenomics-based methods have identified a multitude of new viruses from a variety of environments^1–6^, but there is a recognized need for better representation from soil, plant-associated, and engineered environments^5^. Through their host interactions, prokaryotic viruses can disrupt microbial ecosystem services by reducing or eliminating the hosts responsible^4,5,7,8^, augment host metabolism with Auxiliary Metabolic Genes (AMGs)^9–13^, stimulate gene flow within microbial populations through horizontal gene transfer^14–16^, or alter host immunity through virally-encoded CRISPR arrays^17,18^ that can also drive interviral conflicts^18,19^. While prokaryotic viruses are expected to be host-specific, recent research has provided evidence for non-specific prokaryotic viral infection across taxonomic orders^20^ and even across phyla^5,21,22^.

Current computational methods for matching microbial viruses to their putative hosts vary in accuracy and sensitivity^23^. In comparison to other methods, matching Clustered Regularly Interspaced Short Palindromic Repeats (CRISPR)^24–26^ spacers to protospacers in viral elements yields conservative, relatively accurate predictions^23^. CRISPR spacer to viral protospacer matching has often been utilized in examining virus-host interactions^5,22,23,27–32^ visualized as virus host networks^22,29,30,32^, however, there are few examples of temporal components in network architectures. In one notable example, Martínez Arbas and colleagues^33^ examined the dynamics between integrated Mobile Genetic Elements (iMGEs, which include viruses) in a wastewater treatment system over a year and a half, finding phage-host interactions were tied to community shifts, and observing host CRISPR array evolution over time. The dynamics of virus-host interactions over time in other engineered systems remains an open question.

Landfills are heterogeneous, contaminated, engineered systems of growing environmental and economic importance^34–36^. Contaminants in landfills can be metabolized by bacteria and archaea^35,36^, and, by extension, the viruses that prey on these microorganisms are likely important players in mediating contaminant cycling within landfills. From the perspective of viral diversity and host interactions, landfills remain largely unexamined. Previous work from our group has shown that bacterial and archaeal communities in these systems are diverse and spatially dynamic^37^, but these analyses lacked temporal resolution and did not include an examination of viral communities. Here, we identify the host-associated viral fraction from multiple sites across an active municipal waste site and model the virus-host interactions across two years to determine infection dynamics, host specificities, and interviral conflicts in this dynamic, understudied system.

## Methods

### Sampling

This study makes use of 14 metagenomes, each of which were generated from one of 14 distinct samples collected from an active municipal landfill in Southern Ontario. The sample collection involved three trips, two of which were in July 2016 (six samples collected from five distinct sites) and the third in October 2017 (eight samples taken from eight distinct sites). Examinations of the bacterial and archaeal communities in our July 2016 samples have been previously reported^37^, but no examinations of the virome were performed at that time. Of these samples obtained in July 2016, referred to as the 2016 samples, three were from leachate wells (LW1-3), two were from the same composite leachate cistern that was sampled on two occasions, six days apart (CLC_T1 & CLC_T2), and one sample was obtained from groundwater well adjacent to the landfill (GW1). The relevant October 2017 samples, referred to as the 2017 samples, included samples from the same five sites sampled in 2016, with the addition of samples from one leachate well (LW4), one storm water capture system (SWC1), and one groundwater well (GW3). About 5L of liquid material (leachate or groundwater) was sourced from each of the sampled sites. This material was filtered on-site through a 3 μm glass fiber filter (Pall Corporation, Ann Arbor, MI) and the resulting filtrate was subsequently filtered through a 27 mm diameter 0.1 μm polyethersulfone membrane (Pall Corporation, Ann Arbor, MI) via peristaltic pump filtration until the membrane clogged. Multiple 0.1 μm filters were taken for each site. All filters were stored on dry ice prior to long-term storage at − 80 °C. All samples were handled identically with the exception of CLC_T1, which was filtered through 0.2 µm and 0.1 µm filters sequentially instead of 3 µm and 0.1 µm. For the CLC_T1 sample, biomass on the 0.2 µm filter was used for metagenomic sequencing as there was insufficient biomass on the 0.1 µm filter.

### DNA extractions

DNA was extracted from filters using the PowerMax DNA Isolation Kit (MO BIO Laboratories, Carlsbad, CA) using the default protocol outlined by the manufacturer, with the exception that the filter was cut into small pieces and added to the bead tube in place of soil. Extracted DNA was assessed for quality using the NanoDrop 1000 (Thermo Scientific, Waltham, MA) and quantity using a Qubit fluorometric method.

### Metagenome sequencing, analysis, and host genome binning

Extracted DNA for all 2016 and six 2017 samples was sent to the Joint Genome Institute (JGI, Walnut Creek, CA) for paired-end 150-bp read metagenome sequencing using the HiSeq 2500 (Illumina, San Diego, CA). Read assembly and scaffold annotations, which included CRISPR array annotations employed in downstream analyses, were performed by the JGI in accordance with their most recently described standard operating pipeline^38^. Three of the 2017 samples were sequenced by The Center for Applied Genomics (TCAG, Toronto, Canada). For these metagenomes, quality trimming and assembly followed the published JGI protocol, including bbduk (https://github.com/BioInfoTools/BBMap/blob/master/sh/bbduk.sh) and sickle (https://github.com/najoshi/sickle) for read quality trimming and spades3^39^ (https://cab.spbu.ru/software/spades/) under the -meta flag with kmers 33, 55, 77, 99, and 127 for assembly. Scaffold annotation for all metagenomes was performed by the JGI. Only scaffolds that were greater than or equal to 2.5kbp were considered for subsequent analyses.

All read datasets were mapped individually to each assembled metagenome using Bowtie2^40^. Scaffolds from a single metagenome were binned using three binning algorithms: CONCOCT^41^, MaxBin2^42^, and MetaBAT2^43^. Using DAS Tool^44^, the resulting bins were dereplicated and binned using an iterative consensus-based approach. Genome bins output from DAS Tool were supplied to CheckM^45^ for quality assessment. Taxonomy was assigned to the genome bins using the Genome Taxonomy Database Toolkit^46^ application available on DOE-KBase^47^. Only genome bins that were > 70% complete with less than 10% contamination (defined as medium quality draft Metagenome Assembled Genomes^48^ or better) were considered as putative host MAGs in further analyses.

### Viral element identification and diversity analysis

Scaffolds not included in the putative host MAGs (“unbinned scaffolds”) were supplied as input to the VirSorter^49^ application on CyVerse^50^ with “Virome db” as the reference database and the virome decontamination function turned on. Only viral scaffold predictions from the highest confidence categories (1 and 2 for non-integrated viruses [omitting category 3], and 4 and 5 for proviruses [omitting category 6]) were retained. Unbinned scaffolds were also supplied as input to VIBRANT^51^ using default parameters. Only scaffolds predicted as viral by both VirSorter and VIBRANT were considered for further analysis. We note that while this approach results in robust viral predictions, it potentially overlooks highly novel viruses. These predicted viral scaffolds were clustered through the use of CD-HIT-est^52^ using a global sequence identity threshold of c = 0.95, where c is the number of identical nucleotides over an alignment divided by the full length of the shorter sequence in the alignment. The resulting clusters were considered as viral populations. Only unbinned scaffolds were used for viral detection to prevent CRISPR-array-encoding viral scaffolds that were misbinned within MAGs from having matches to themselves in the virus-host linking workflow described below. All representatives of viral populations were used as input to vConTACT2^53^ in order to assess how similar viral communities are across samples and between our two timepoints. Gene-sharing networks created from vConTACT2 were visualized using Cytoscape ver. 3.8.0 (https://cytoscape.org/)^54^.

### Virus-host linking

Viral elements were linked to their hosts through matching host CRISPR array spacers to protospacers found in the viral elements that were agreed upon by VirSorter and VIBRANT. CRISPR spacers in genome bins were BLASTn^55^ searched against all viral elements, with hits retained only if they had up to one mismatch, a query coverage of ≥ 90% and an E-value ≤ 10^−4^. The results of these BLASTn searches were visualized as prokaryotic virus-host networks using Cytoscape ver. 3.8.0. Recent analyses have shown that oligonucleotide and abundance profiles are less reliable for host detection relative to CRISPR- and provirus-based analyses^23^, thus we did not use co-binning of unintegrated viral scaffolds as valid links for putative virus-host linkages.

### Community membership between networks

We first used dRep^56^ to compare all 2016 MAGs to the MAGs reconstructed from 2017 datasets, regardless of whether MAGs were included in the networks. We then used CD-HIT-est (global sequence identity threshold of c = 0.95) to compare all 2016 viral elements to those predicted from 2017 datasets, again irrespective of whether any of these viral elements were present in the networks. To understand whether the hosts and viruses observed in the 2016 network were detected and included in the 2017 network, we used dRep and CD-HIT to dereplicate the network-associated hosts and viruses, respectively. For any MAGs that were shared between the 2016 and 2017 networks, we examined changes in their detected CRISPR arrays using command-line tools and CRISPRCasFinder^57^.

### Interviral conflicts

All high confidence viral scaffolds from VirSorter and VIBRANT, prior to clustering with CD-HIT-est, were cross-checked against the CRISPR array annotations provided by the JGI. The spacers from putatively virus-encoded CRISPR arrays were used as input into a BLASTn search (hits retained only if they had up to one mismatch, a query coverage of ≥ 90% and had an E-value ≤ 10^−4^) against a database of all high confidence viral scaffolds agreed upon by VirSorter and VIBRANT. Matches between spacers from two distinct viral CRISPR arrays were not considered genuine interviral conflicts. To identify relevant proviruses, putative viral elements containing CRISPRs were BLASTn searched against all genome bins. In accordance with previous literature, putative provirus detection was considered with a bit score threshold of 50, an E-value threshold of 10^−3^, and a minimum alignment length of 2500 bp with at least 70% average nucleotide identity over the length of the alignment^28^. If 90% or more of a scaffold in a host genome bin aligned with a putative viral scaffold, this was considered to be indicative of a viral scaffold being co-binned with host scaffolds rather than being integrated in a host scaffold as a provirus^28^. All putative proviruses identified through the aforementioned approach were then confirmed using Prophage Hunter^58^ and PHASTER^59^ where if either tool detected a proviral element, said element was incorporated into downstream analyses. The CRISPR arrays and CRISPR-Cas systems of putative proviruses were further validated using CRISPRCasFinder. For CRISPR-encoding proviruses, we manually inspected whether the host targeted a specific viral element via the CRISPR array provisioned by its provirus, or via a different CRISPR array within its genome.

In the case of the 187-spacer CRISPR array, we read-mapped reads from the original assembly to the scaffold to confirm connection of this array to viral marker genes. Two possible consensus sequences were identified at the 3’ end of the CRISPR array, with the higher depth option connecting to the viral segment of the scaffold. The second, lower abundance sequence may represent an alternate current host or the original bacterial host for this array.

### Hyper-targeted virus analyses

The CRISPR-spacer targets of all four viral elements in 2016 that were hyper-targeted by members of both the 2016 and 2017 hosts in our networks were manually examined in Geneious version 2021.1.1 (https://www.geneious.com/prime/)^60^. Detection of the Open Reading Frames (ORFs) in the viral elements and their initial annotations were performed during the JGI’s annotation as described above. Predicted ORFs that were not annotated by the JGI’s pipeline were manually annotated using hmmer3^61^ (http://hmmer.org/) and Hidden Markov Models from the Virus Orthologous Groups database (https://vogdb.org/, release number vog206).

### Examining the potential for viral infection across the domain and phylum levels

To further validate putative cross-phylum and cross-domain infecting viral elements, we performed a series of additional analyses: the quality of the MAGs predicted to target the implicated viral elements was considered, the length of the scaffolds encoding CRISPR arrays that targeted the implicated viral elements was examined, the CRISPR arrays from these scaffolds were compared between hosts targeting the same viral element, and the gene content and taxonomic affiliation of these scaffolds were assessed to aid chimera and misbin detection. The legitimacy of virus-host interactions was assessed on a case-by-case basis with an emphasis on taxonomic congruence between MAGs and their CRISPR array-encoding scaffolds (Supp. Table 1).

### Computing platforms

The bulk of the computational analyses performed were run on an in-house server with an Ubuntu 18.04 operating system. CyVerse was used specifically to run VirSorter, DOEKBase was used specifically to run GTDB-tk and vConTACT2, and for three of our 2017 metagenomes that were sequenced outside the JGI, Compute Canada’s Graham cluster was used for the spades3 assembly step in the metagenomics workflow.

## Results

### Identification of MAGs and viral elements

We sampled microbial biomass from a municipal landfill in Southern Ontario in July 2016 and October 2017. The sample sites in 2016 included three distinct leachate wells (LW1-3), a groundwater well adjacent to the landfill (GW1), and two temporal samples from the same leachate collection cistern, collected one week apart (CLC_T1 & CLC_T2). The 2017 sample series included samples from each of the five 2016 sites, with the addition of a fourth leachate well (LW4), an additional groundwater well (GW3), and a storm water catchment collection system (SWC1).

DNA extraction from sample biomass followed by metagenomic sequencing, assembly, and binning of the 2016 data yielded 688 MAGs that were > 70% complete (70.13% to 100.00%, average completion 83.72%) and had < 10% contamination (0.00% to 9.54%, average contamination 1.83%) (Table 1). Collectively, these bins are considered, at minimum, medium quality draft Metagenome Assembled Genomes^48^. The 2017 data yielded 1064 MAGs that were > 70% complete (70.09% to 100.00%, average completion 85.68%) and had < 10% contamination (0.00% to 9.99%, average contamination = 2.88%). Viral detection using VirSorter^49^ and VIBRANT^51^ followed by dereplication with CD-HIT-est^52^ predicted 17,057 and 18,877 viral elements from the 2016 and 2017 data, respectively (Table 1). Examination of viral diversity across sample sites (Supplemental Fig. S1) and years (Supplemental Fig. S2) identified highly diverse viral communities with significant similarities across space and time.

**Table 1:** Summary statistics for metagenomes, MAGs, and viral scaffolds from the sampling sites.

Sample name	Read Count	Scaffolds >= 2.5kbp	MAGs^1^	% scaffolds in MAGs	# viral scaffolds^2^
CLC_T1 (2016)	199,293,412	92,890	71	18.25	3948
CLC_T2 (2016)	187,799,476	110,160	134	28.62	3368
GW1 (2016)	170,612,252	85,861	161	20.99	856
LW1 (2016)	177,231,300	93,614	72	12.6	7120
LW2 (2016)	200,095,936	108,918	169	31.85	2801
LW3 (2016)	199,991,448	90,376	81	14.28	5117
CLC (2017)	258,387,248	90,316	143	37.91	1532
GW1 (2017)	344,037,992	174,133	273	16.39	2919
GW3 (2017)	125,101,280	19,781	54	33.97	66
LW1 (2017)	104,844,754	59,502	54	14.36	3635
LW2 (2017)	270,424,804	90,072	160	34.45	2071
LW3 (2017)	318,700,912	184,907	149	13.9	12,241
LW4 (2017)	258,469,368	89,271	171	21.36	2728
SWC1 (2017)	142,415,766	33,875	60	29.19	152

^1^Quality filtered bins with > 70% completion, < 10% contamination).

^2^Viral scaffolds detected from unbinned scaffolds (prior to CD-HIT clustering).

**Figure 1:**
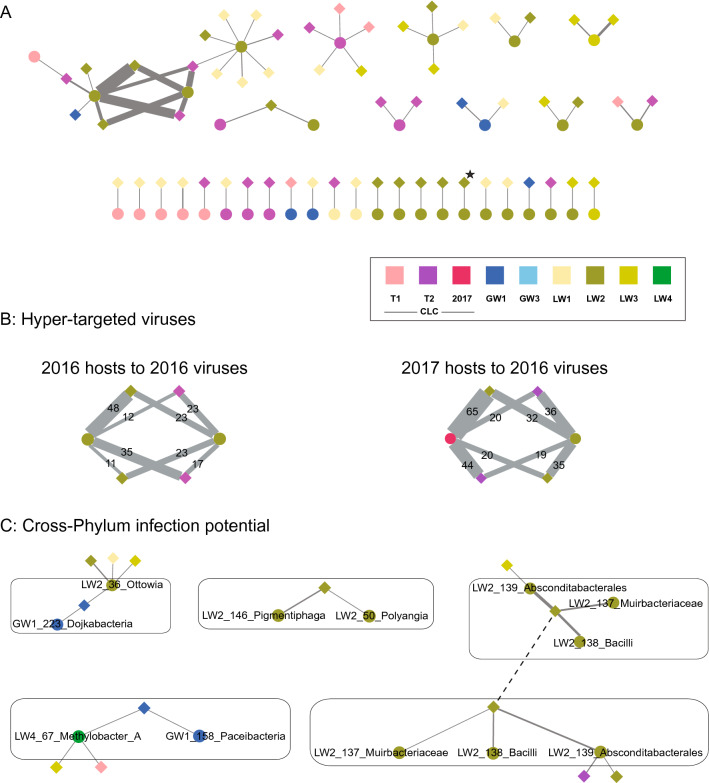
(**A**) Virus-host interactions in a Southern Ontario landfill mapping 2016 hosts and 2016 viruses. CRISPR array spacers from the MAGs (circles) were mapped against the viral elements (diamonds) predicted by VirSorter and VIBRANT to establish virus-host linkages (connecting edges). The network is colored by the geographic sampling location from which the MAG or viral element originated, with the width of the edges proportional to the number of spacer-protospacer matches supporting the connection. A viral element encoding a Diversity Generating Retroelement (DGR)^62^ is denoted by a star. MAG identifier and lowest level of taxonomy as determined by GTDB-Tk^46^ are noted in Supplemental Fig. S3. The other three networks are presented in full in Supplemental Figs. S4–S6. (**B**) Hyper-targeting dynamics between 2016 (left) and 2017 (right) hosts against the 2016 viral elements, with the number of spacer-protospacer matches supporting the connections. (**C**) Viral elements and hosts predicted to be involved in cross-phylum infections across all virus-host networks. The two viral elements connected by a dashed edge are predicted to be the same viral element in common between 2016 and 2017.

**Figure 2:**
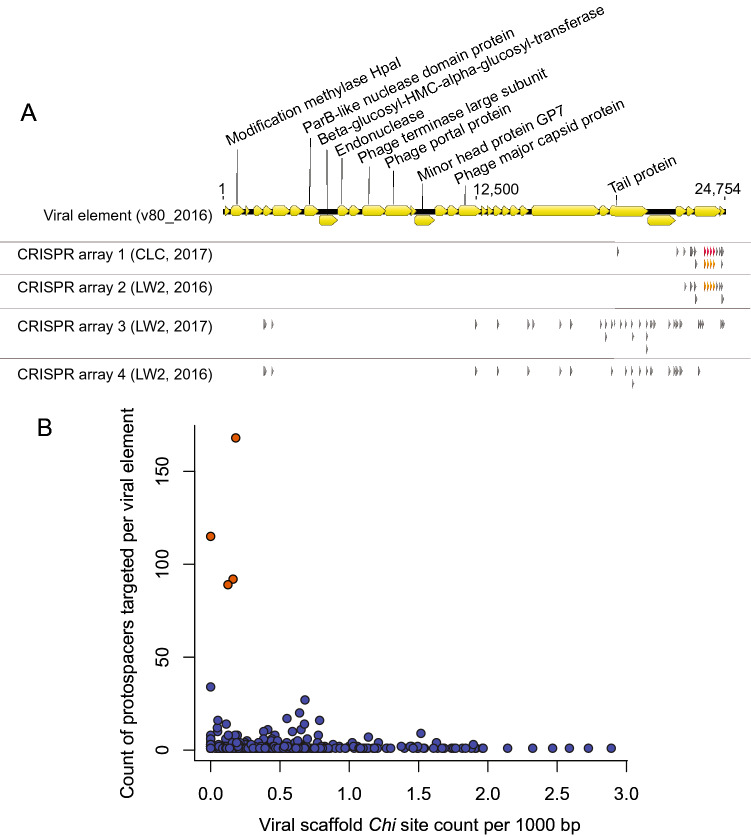
(**A**) Genetic features targeted by host CRISPR arrays on a hyper-targeted viral element. The coding sequences’ annotations for one hyper-targeted viral element (V80_2016) with corresponding CRISPR spacer mappings are displayed. In the targeting profile of a single host scaffold, a non-grey triangle represents a single spacer that had multiple matches to regions of the viral element. When shared, these single spacers are colored the same between distinct host targeting profiles. CRISPR spacer mapping and visualization was conducted using Geneious^60^. Host scaffold accessions are as follows: CRISPR array 1: Ga0265294_10000314; CRISPR array 2: Ga0172382_10051677; CRISPR array 3: Ga0265293_10000924; CRISPR array 4: Ga0172382_10020296 & Ga0172382_10071763 (two arrays within one host MAG, only one is responsible for hyper-targeting). (**B**) *Chi* site counts in viral elements (in *Chi* site per 1000 bp) compared to number of times a viral scaffold was targeted by a host across all virus-host networks. Hyper-targeted viral elements are highlighted in orange.

### Prokaryotic virus-host interactions

36% of our 2016 MAGs and 42% of our 2017 MAGs encode CRISPR arrays. CRISPR spacers were excised from CRISPR arrays and matched to viral protospacers allowing one mismatch. Virus-host interaction networks were derived for all host CRISPR spacer to virus protospacer combinations (2016 hosts – 2016 viruses; 2017 hosts – 2017 viruses; 2016 hosts – 2017 viruses; and 2017 hosts – 2016 viruses; Table 2, Supplemental Tables S2–S5) to explore host-virus dynamics and adaptive immunity over time. We identified between 37–90 host MAGs implicated in a network, with up to 173 unique links between hosts and viral scaffolds (unique links = one MAG to one viral element, regardless of number of spacer connections; Table 2, Supplemental Table S6). Viral elements and predicted hosts were frequently from different sample sites (Fig. 1, Supplemental Figs. S3–S6, Supplemental Table S7). Across all networks, 28–44% of host MAGs were connected to multiple viral scaffolds, targeting from two to nine predicted viral elements (see Supplemental Tables S2–S5 for specific MAG-viral element connections). 1–10% of viral elements were predicted to infect two or more distinct hosts.

**Table 2:** Virus-host networks summary. Virus-host interactions are based on host MAG CRISPR spacers and viral element protospacer connections. Unique links = 1 MAG to 1 viral element, regardless of number of spacer connections.

Network	Host MAGs	Viral scaffolds	Unique links
2016	37	59	66
2017	90	161	173
2016-host-to-2017-viruses	39	79	80
2017-host-to-2016-viruses	87	125	139

**Figure 3:**
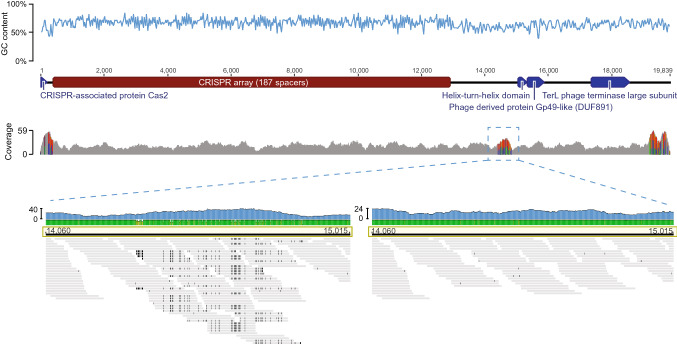
Assessment of 187 spacer encoding viral scaffold. From top to bottom: GC content along the length of the scaffold (total length 19,839 bp); annotation map of relevant genes; depth of read coverage and heterogeneity. The variable region between 14K-15K is marked with a blue box. Full read mapping on the left demonstrates two potential sequences for this region. On the right, the higher abundance path is shown. This image was made using Geneious^60^ and IGV^63^.

### Temporal community continuity

As microbial communities in this system are poorly conserved across sites^37^, we were interested in examining the temporal stability in the host-viral interactions as well as the microbial community as a whole. We used dRep^56^ to cluster the reconstructed MAGs into populations. In the 2016 dataset, 525/688 MAGs passed dRep’s quality filtering. Of these, 232 (~ 44%) were represented in the 2017 MAGs dataset. For the 2017 dataset, the 232 shared MAGs comprise 28% of the 824/1064 MAGs that passed dRep filtering (see Supplemental Fig. S7 for a visual summary of these overlaps). Viral elements were clustered with CD-HIT, which resulted in 3198 viral elements represented in both datasets, comprising 18.8% and 16.9% of the viral elements annotated in the 2016 and 2017 metagenomes, respectively (Supplemental Fig. S7).

We next investigated community composition between our temporally segregated networks. There were fewer conserved viral elements and MAGs within the host-virus networks compared to the overall datasets. For the viral elements, 19/59 viral elements (32.2%) from the 2016 network were represented in the entire set of 2017 virus annotations. Only 5/59 viral elements (8.5%) had near-identical viral elements in the 2017 virus-host network. For the hosts, of the 36 2016-networked MAGs that passed dRep’s quality filtering, 16 (~ 44%) were represented in the 2017 MAG dataset as a whole, and only three (~ 8%) were present in the 2017 network (Supplemental Fig. S7).

Two virus-host pairs were shared between the 2016 and 2017 networks (i.e., viral elements clustered at 95% global sequence ID, hosts collapsed by dRep). Each of the two host MAGs in 2017 shared no spacers in their encoded CRISPR arrays with their 2016 counterparts, meaning each host targeted the same viral element with different CRISPR spacers in each year. In one of the host MAGs, the CRISPR arrays shared an exact match in a sequence flanking their respective CRISPR arrays (Supplemental Fig. S8). Based on sequence similarity to the direct repeat element found in both CRISPR arrays, this flanking sequence may contain a CRISPR spacer and a partially degraded repeat^64^, which is evidence for a shared CRISPR array origin within this population even in the current absence of shared spacer sequences. Note all subsequent work did not use dereplicated MAGs to preserve differences in CRISPR array structure.

### Cross-phylum/cross-domain infection

In the networks, 14 viral elements were predicted to infect multiple hosts that are from different phyla (8) or domains (6) (Supplemental Table S1). Following curation based on length of CRISPR-array containing scaffolds, quality of MAGs, and taxonomic consistency between MAGs and CRISPR-array encoding scaffolds, we predict that four cross-phylum viral infections are legitimate, one of which was identified in two networks. No cross-domain interactions passed our quality screen (see Supplemental information for more details).

In the 2017 network, two viral elements were implicated in connections that passed subsequent quality control (Supp. Table 1). One viral element (V2_2017) showed infection potential against hosts from three distinct phyla: the class Bacilli (LW2_138, phylum Firmicutes), the family Muirbacterieaceae (LW2_137, phylum Muirbacteria), and the order Absconditabacteriales (LW2_139, phylum Patescibacteria), all of which, including the viral element itself, were identified at the same sample site. These same three MAGs also shared a common viral element in the 2017-hosts-to-2016-viruses network (V154_2016). Based on 95% sequence identity clustering, the 2016 viral element is predicted to be the same as the 2017 viral element (V2_2017). The second 2017 cross-phylum interaction involves a member of the genus *Ottowia* (LW2_36, phylum Proteobacteria) and a member of the class Dojkabacteria (GW1_223, phylum Patescibacteria) which were both predicted to be infected by a single viral element (V102_2017).

In the 2017-host-to-2016-viruses network, we saw two additional instances of viral elements with potential to infect across phyla. One viral element (V36_2016) showed potential to infect hosts from the genus *Pigmentiphaga* (LW2_146, phylum Proteobacteria) and the class Polyangia (LW2_50, phylum Myxococcota). Finally, one viral element (V42_2016) was predicted to infect GW1_158_Paceibacteria (phylum Patescibacteria) and LW4_67_Methylobacteria (phylum Proteobacteria). Interestingly, CRISPR arrays targeting this viral element share 56 identical spacers, suggesting a horizontal gene transfer event of this array between these two hosts.

We observed no instances of viral elements with potential to infect across phyla in the 2016-host-2017-viruses network and none of the observed interactions in the 2016 network passed quality control.

### Diversity generating retroelements encoded by viruses

Diversity Generating Retroelements (DGRs) may increase a virus’ breadth of infection^62^. Leveraging the results of a previous meta-analysis in which a subset of our datasets were included^62^, we report 92 DGR targets in our 2016 virus data and 30 from six of nine 2017 datasets. In total, eight viral elements that are involved in our networks were predicted to contain DGRs (star in Fig. 1, Supplemental Figs. S3–S6), but none of these viral elements were predicted to infect across different phyla or domains.

### Hyper-targeting of viral elements

Five viral elements were hyper-targeted by host MAG CRISPR arrays, where hyper-targeting was defined as having ≥ 20 protospacers targeted by a single host’s set of CRISPR spacers. Hyper-targeted viral elements were not observed in the 2017 or 2016-host-to-2017-virus networks. One 2016 viral element was hyper-targeted by 2017 host MAGs, where the viral element had not been targeted by the 2016 hosts. The other four viral elements were hyper-targeted in both the 2016 and 2017-host-to-2016-viruses networks (V111_2016, V80_2016, V168_2016, and V65_2016), allowing examination of CRISPR arrays over time (Fig. 1B). For these four viral elements, two host MAGs were responsible for the observed hyper-targeting in each network, and the CRISPR arrays responsible for the hyper-targeting were highly conserved over their overlapping region between the two temporal samples. The MAGs involved in hyper-targeting had different taxonomies between the 2016 and 2017 hosts, with two members of the Acholeplasmataceae (Firmicutes) hyper-targeting the four viral elements in 2016, and two members of the Bacteroidales (Bacteroidota) hyper-targeting the same viral elements in the 2017-hosts-to-2016-viruses network (See supplemental data for details). It was not clear whether this is due to lateral gene transfer of CRISPR-array-encoding regions or mis-binning of CRISPR-encoding scaffolds, so we focused instead on array structure and nature of the hyper-targeting on the viral elements.

Each of the four hyper-targeted viral elements was targeted more times by the 2017 hosts (members of the Bacteriodales) relative to the 2016 hosts (members of the Acholeplasmataceae), though some of these connections were not reinforced by at least 20 host spacer to viral protospacer matches and thus were not considered hyper-targeting. The highest degree of targeting (against V111_2016) was 65 CRISPR spacer to viral protospacer connections, compared to 48 connections to the same viral element in the 2016 network. CRISPR spacers aligned to the hyper-targeted viral elements showed preferential targeting of specific regions within these elements (Fig. 2A). The two regions most strongly targeted were tail protein coding sequences and hypothetical coding sequences (Fig. 2A). In some instances host targeting of viral elements appeared biased to one end of the viral elements (Fig. 2A, Supplemental Fig. S9).

We compared host CRISPR array-encoding scaffolds to assess the nature of these shared connections. For 3 of 4 host MAGs, connections to hyper-targeted viral elements stemmed from a single CRISPR array on the genomes, even in cases where multiple CRISPR arrays were encoded. For one pair of hyper-targeting CRISPR arrays, the shorter 2016-host-encoded array was a subset of the longer 2017-host-encoded array, without flanking regions to define a complete array and compare array adaptation over time. For the second pair of CRISPR array-encoding scaffolds, the 5’ end and flanking region of the CRISPR arrays was captured on both scaffolds. There were no additional spacers incorporated into the CRISPR array over the ~ year between sampling events, which may suggest that the array is inactive given CRISPR arrays can change over short time scales^65^. We note that three of the four MAGs involved in hyper-targeting are among the top ten most abundant network-associated MAGs across all metagenomes described here, with one (LW2_114_2017, order Bacteroidales) as the most abundant MAG in the dataset (average coverage of 877).

We predicted *Chi* sites in all viral elements that were targeted by hosts to assess if hyper-targeting was correlated with a depletion in *Chi* sites, which is associated with increased spacer recruitment^66^. All hyper-targeted viral elements were among those with the lowest proportional counts of *Chi* sites (mean = 0.48 and median = 0.12 per 1000 bp for all targeted viral elements) with counts for hyper-targeted viral elements being 0.16, 0.12, 0.18, and 0 [mean: 0.12, median: 0.14] Fig. 2B).

### Virally-encoded CRISPR arrays convolute virus-host networks

Viruses have been shown to encode CRISPR arrays as well as Cas proteins, with hypotheses that include these systems acting as defense mechanisms preventing other viruses from infecting the same host^18,19^. We explored the potential for interviral conflicts using the full set of viral predictions prior to clustering with CD-HIT in order to preserve CRISPR array variation between closely-related viral elements. In our 2016 data, 70 (0.41%) viral elements were predicted to encoded CRISPR arrays, with no shared CRISPR spacers between viral elements. These arrays contained between 2–187 spacers (average spacer count per array ~ 7, 6 arrays contained > 10 spacers). The current longest CRISPR array is 587 spacers long, from *Haliangium ochraceum* strain DSM 14,365^67^. To our knowledge, the 187-spacer viral CRISPR array is the longest viral CRISPR element that has been predicted to date. This element also contains a *cas2* gene, which is further evidence of functionality, but is uncommon, as most phage CRISPR systems do not encode spacer acquisition machinery^6,68^ (Fig. 3). VIBRANT predicted that this genetic element was a non-integrated virus and Prophage Hunter did not identify any proviruses within the element. Based on read mapping, the scaffold containing the 187-spacer CRISPR array showed high heterogeneity in a region between the CRISPR array and viral marker genes (Fig. 3). Curation of reads mapping to this region confirmed appropriate depth of coverage for the consensus sequence linking the array to the viral genes (Fig. 3). In our 2017 data, 91 (0.48%) viral elements were predicted to encode CRISPR arrays. There were 8 instances of shared CRISPR spacers between CRISPR-encoding viral elements. The 2017 virally-encoded CRISPR arrays contained between 2–21 spacers (average spacer count per array ~ 4, 4 arrays contained > 10 spacers).

Spacers encoded in 2016 virus CRISPR arrays were predicted to target 32 viral elements in the unclustered 2016 virus dataset; whereas spacers encoded in 2017 virus CRISPR arrays were predicted to target 44 viral elements in the unclustered 2017 dataset. These may represent latent interviral conflicts which would need to be co-located in a cellular host for conflicts to be active.

Within our host-virus networks, viral elements encoding CRISPR arrays were predicted to be proviruses in 3 hosts (see supplemental information for details). In all cases, connections in the networks between provirus-encoded CRISPR arrays and non-integrated viral elements were based on shared CRISPR spacers in CRISPR arrays on both viruses rather than a spacer-protospacer match. When CRISPR spacers are integrated into an array, the protospacer-adjacent motifs (PAMs) are not incorporated. As a result, CRISPR spacers are not active targets for Cas nucleases^69^. In one case, both the host-encoded and provirus-encoded CRISPR arrays target the same non-integrated viral elements. In another case, the viral element contains two distinct CRISPR-Cas systems, both of which are shared in the provirus and non-integrated virus versions of this element.

## Discussion

Using a metagenomics-based approach, we were able to resolve prokaryotic virus-host interactions and infection networks from samples collected from a municipal landfill and adjacent aquifers approximately one year apart. Cellular populations were more stable than viral elements, with ~ 44% and ~ 19% conserved between the two years, respectively. Infection networks were more dynamic, with lower continuity of MAGs and viral elements between the 2016 and 2017 networks compared to the baseline communities.

It was common for a viral element and its predicted host to be from different sample sites. Planktonic viruses were expected to pass through the 0.1 μm filters used to collect biomass, meaning most of the prokaryotic viruses detected were captured from active infections and indicate presence of the host even if it was not detected as a high-quality MAG from that site’s metagenome. Hosts in our networks were connected to 1–9 viral elements, potentially representing a large infection burden for some populations. Alternatively, multiple viral elements infecting the same host may be genomic fragments of the same viral genome. In our temporal comparison, the 2017-host-to-2016-virus network was not larger than the 2017-only network, despite a hypothesis that hosts from 2017 would have established immunity to 2016 viruses via earlier infections.

We predicted four instances where viral elements infect members of multiple phyla. Cross-phyla infection potential for viruses has been predicted in silico before^5,22^, and also confirmed in a single study for four bacteriophages isolated from Lake Michigan^21^. There are numerous adaptations a viral element would require in order to successfully infect multiple hosts, none of which were examined here. These adaptations would be required for crucial steps of the infection process such as host target receptor recognition and evasion of host immunity, the former of which would likely lower infection efficiency on the original host^70^. While we have no evidence against four putative cross-phylum interactions, laboratory confirmation is a necessary requirement to substantiate these predictions.

Diversity generating retroelements (DGRs) are genetic elements that enhance mutation and evolution in microbes, including viruses^62^. DGRs were shown to be enriched in landfills^62^, possibly owing to landfills’ extreme heterogeneity and concentration of solvents and other stressors increasing the evolutionary pressures on microbes, thus selecting for DGRs to enhance microbial adaptation. DGRs can also facilitate diversification of virus host-attachment proteins, where viruses that encode DGR targets within their host attachment protein encoding genes show a broader host range^62^. In our networks, none of our putative broadly-infecting viral elements encode any detectable DGRs, and viral elements with DGRs were associated with only one host or, at most, two hosts from the same family. DGRs thus do not appear to be strong factors in influencing the instances of broad viral host range in the networks from this study.

A striking feature of our networks were the hyper-targeted viral elements, with up to 65 unique connections between a host’s CRISPR spacers and single viral elements. Hypothetical protein coding regions on the viral elements were highly targeted, suggesting that the proteins encoded in these regions are important for viral propagation. A bias of targeting at one end of a viral element was observed in some cases which may be due to some CRISPR-Cas systems preferentially recruiting spacers from the regions of viral DNA first injected into the host cell, as this provides better immunity than spacers from late-injected regions^71^. The hyper-targeting of viral elements may have evolved due to an on-going arms-race between host and virus which builds a history of immunity over time; either from a strong priming response in the CRISPR-Cas system, which results in recruitment of multiple spacers once activated^72,73^; or because the viral element is deficient in *Chi* sites, which are molecular benchmarks for preventing DNA cleavage by the repair complex^66^. Hyper-targeted viral elements in the 2017-hosts-to-2016-viruses network showed higher levels of hyper-targeting than in the 2016 network. The hyper-targeting of the same four viral elements by hosts in 2016 and 2017 suggests a long history of infection dynamics. The hyper-targeted viral elements had a relatively low proportion of *Chi* sites when compared to viral elements with weaker targeting (Fig. 2B). The viral elements with the highest proportion of *Chi* site counts were not strongly targeted in our networks.

CRISPR arrays encoded on viral elements were found in a small fraction of predicted viral elements (0.45%). Virus-encoded CRISPR arrays and CRISPR-Cas systems have been identified before^6,17–19,68,74–77^ and are involved in crippling host viral defense systems^17^, regulating host transcription and translation^6^, CRISPR-Cas system inhibition^18^ and interviral conflicts^18,19^. In the case of interviral conflicts, a CRISPR-encoding virus, integrated as a provirus, can provide its host with immunity against other viruses, akin to a superinfection exclusion system^18^. Our datasets contained a putative viral element (V170_2016) that encodes a CRISPR array with 187 spacers and a *cas2* gene, the longest virally-encoded CRISPR array to date. Although this viral element was not targeted by any bacterial or archaeal hosts in our networks, CRISPR spacers within its array did target two other viral elements in the 2016 data, neither of which were predicted to encode CRISPR arrays themselves. Due to *cas2* gene being the last gene on one end of the scaffold, along with limitations on the accuracy of prophage boundary detection tools, it is possible that this scaffold contains a bacterial CRISPR array adjacent to an integrated prophage.

In our networks, three proviruses containing CRISPR arrays targeted their own planktonic population of viruses. These connections were due to CRISPR spacers that are common to the arrays of both the proviruses and their non-integrated virus equivalents. Due to the requirement of a Protospacer-adjacent motif (PAM) for virtually all Cas nucleases^69^, spacer-to-spacer matches wouldn’t result in immunity unless the hypothetical nuclease involved in target cleavage was PAM agnostic or PAM permissive. Two connections in our networks were exclusively due to putative provirus CRISPR-arrays matching to their non-integrated virus equivalent’s CRISPR arrays, however, the presence of the provirus itself indicates that the non-integrated virus equivalent is able to infect the host.

Within this engineered system, virus-host interactions are substantially more complicated than a 1-virus-1-host model. Cross-phylum infections, hyper-targeting, interviral conflicts, and provirus-encoded CRISPR arrays were observed in a single system, suggesting these interactions may be more common than currently understood. We hope that in future, these interactions are curated on a case-by-case basis as we have done here, in order to expand our understanding of virus-host interactions in natural environments. We note that this wealth of insight derived from a single landfill, and suggest this category of highly heterogenous engineered environments merits further study of microbial communities and virus-host interactions.

## Supplementary Information

Supplementary Information 1.

Supplementary Information 2.

## Data Availability

The assembled and annotated Southern Ontario 2016 metagenomes are deposited on IMG with the following IMG Genome IDs (Taxon Object IDs): 3300014203 (CLC1_T1), 3300014206 (CLC1_T2), 3300014204 (LW1), 3300015214 (LW2), 3300014205 (LW3), and 3300014208 (GW1) (https://img.jgi.doe.gov/cgi-bin/m/main.cgi). The assembled and annotated Southern Ontario 2017 metagenomes are deposited on IMG with the following IMG Genome IDs (Taxon Object IDs): 3300014203 (CLC), 3300014206 (GW1), 3300028028 (GW3), 3300015214 (LW1), 3300028603 (LW2), 3300014208 (LW3), 3300014208 (LW4), and 3300014208 (SWC1). All Metagenome Assembled Genomes connected in virus-host networks are available on NCBI under the BioProject PRJNA823399 and accessions JALNVE000000000-JALOCK000000000 (https://www.ncbi.nlm.nih.gov/bioproject/PRJNA823399).
