# Supplementary material for: CRISPR-resolved virus-host interactions in a municipal landfill include non-specific viruses, hyper-targeted viral populations, and interviral conflicts
Source: Sci Rep. 2023 Apr 5;13:5611. doi: 10.1038/s41598-023-32078-6 (PMC10076291; doi:10.1038/s41598-023-32078-6)
Supplement: Supplementary file 1 — Supplementary Information 1. [file 41598_2023_32078_MOESM1_ESM.pdf]

CRISPR-resolved virus-host interactions in a municipal landfill include non-specific viruses, hyper-targeted viral populations, and interviral conflicts

Nikhil A. George[a], Laura A. Hug[a*]

Supplemental Results and Discussion

Prokaryotic virus-host interactions

The 2016 host-virus network included 37 unique host genome bins with 66 unique links to their predicted prokaryotic viral elements (Supplemental Figure 3). In the 2017 network, 90 unique host MAGs had a total of 173 unique links to predicted prokaryotic viral elements (Supplemental Figure 4). In our 2016-host-to-2017-virus network (Supplemental Figure 5) we observed 80 unique links between prokaryotic viral elements and their predicted hosts (compared to 66 in the 2016 network [Figure 1]) and 39 unique host genome bins (compared to 37 in the 2016 network). In general, this network was simpler than the 2016-2016 or 2017-2017 networks. In the 2017-host-to-2016-virus network (Supplemental Figure 6) we observed 139 unique links between prokaryotic viral elements and their predicted hosts (compared to 173 in the 2017 network [Supplemental Figure 4]) and 87 unique host genome bins (compared to 90 in the 2017 network). The virus-interacting MAGs in the 2016 network ranged in completion from 70.46% to 100.00% (average completion 91.39%) and ranged in contamination from 0.00% to 9.47% (average contamination 2.45%). The virus-interacting MAGs in the 2017 network ranged in completion from 70.30% to 100.00% (average completion 90.98%) and ranged in contamination from 0.00% to 9.99% (average contamination 3.25%). The virus-interacting MAGs in the 2016-host-to-2017-virus network ranged in completion from 70.36% to 100.00% (average completion 89.61%) and ranged in

contamination from 0.00% to 7.27% (average contamination 2.43%). The virus-interacting MAGs in the 2017-host-to-2016-virus network ranged in completion from 70.53% to 100.00% (average completion 89.45%) and ranged in contamination from 0.00% to 9.99% (average contamination 3.45%).

In the 2016 network, six of 59 viral elements were predicted to infect two or more distinct hosts. In the 2017 network, eleven of 161 viral elements were predicted to infect two or more distinct hosts. In the 2016-host-to-2017-viruses network, one of 79 viral elements were predicted to infect two or more distinct hosts. In the 2017-hosts-to-2016-viruses, thirteen of 125 viral elements were predicted to infect two or more distinct hosts.

Curation of cross-phylum and cross-domain virus-host interactions

14 viral elements were predicted to infect multiple hosts that are from different phyla (8) or domains (6), in a total of 10 interactions (Supplemental Table 1). Curation took the following requirements into consideration:

1) Host genome quality – following our baseline thresholds of >70 completion and <10 contamination, all 10 interactions pass
2) CRISPR-encoding scaffolds' taxonomic annotations from JGI scaffold annotations[1] and/or the Contig Annotation Tool[2] were required to be consistent with GTDB-tk-based MAG taxonomy[3] – six of the remaining interaction sets had erratic scaffold annotations (four remaining)
3) CRISPR-encoding scaffolds were required to be minimum 4,000 bp long – no additional interactions were eliminated. Four interactions pass quality curation.

Only in the case of GW1_158 (class Paceibacteria) and LW4_67 (genus *Methylobacter*) did two MAGs share any spacers in the CRISPR array responsible for the targeting of their mutually shared virus. Notably, GW1_158's array contained 57 spacers and LW4_67's contained 78, but 56 of these spacers were shared with 100% nucleotide identity. The scaffold of LW4_67 relevant to this analysis is the longer of the two (15,46 includes an intact Type I CRISPR-Cas system[4], and numerous coding sequences with predicted species-level taxonomy that is in accordance with the GTDB-tk annotated taxonomy of this MAG. While the scaffold of GW1_158 relevant to this analysis only has a length of 4,111 bp, this scaffold also has predicted species-level taxonomy, according to the Contig Annotation Tool, that is in accordance with the GTDB-tk annotated taxonomy of this MAG. We thus predict the shared spacers are the result of a possible horizontal gene transfer of a CRISPR array via a mobile genetic element[5], but could also be due to a misbin, a possibility that cannot be refuted for such a short fragment. In all other cases, no spacers were shared, reducing the likelihood that the predicted shared viral targeting is underlaid by lateral gene transfer or mis-binning (Supplemental Table 1).

Three of the refuted cross-domain interactions all include the same Halobacterota bin (CLC_33_Methanoculleus_2017), and three different bacterial partners – two Cloacimonadota and one Spirochaetota (CLC_82_Cloacimonadaceae_2017, LW3_15_Cloacimonadaceae_2017, and CLC_92_Sphaerochaeata_2017). CLC_33_Methanoculleus_2017 contains a CRISPR array on a relatively short scaffold (3,667 bp) that targets two different viral scaffolds, each of which is targeted by a distinct Cloacimonadota MAG. This same *Methanoculleus* MAG encodes a second CRISPR array on another short scaffold (3,710) that targets a different viral scaffold that is also targeted by a Sphaerochaeata MAG. This may be a legitimate signal, or the result of multiple

orphaned CRISPR arrays or mobile elements binning into this genome. We note the observation as worth monitoring in other systems and future samples from this system.

Within the cross-phylum interactions, there was no consistent pattern of phyla involved, including connections between Bacteroidota-Firmicutes, Chloroflexota-Firmicutes, Patescibactera-Proteobacteria, and others (See Supplemental Table 1).

We anticipate that, as computational methods for associating hosts and viral elements improve, there will be more instances of viruses that show potential to infect across relatively high taxonomic levels. We hope that rather than defaulting to these interactions being workflow artifacts, or considering them as ground truths, they are examined critically on a case-by-case basis.

Hyper-targeting of viral elements

In the 2016 network, a member of the family Acholeplasmataceae_A (LW2_3) showed the strongest targeting, with 48 CRISPR spacer matches to a single viral element (v111_2016) (Figure 1B, Supplemental Figure 3). In the 2017 host-to-2016-viruses network, while the number of unique virus-host links was less than in the 2017-only network (139 compared to 173 in our 2017 network [Table 1]), we now also observed 2017 hosts with the ability to hypertarget viral elements (Figure 1B). It is notable that these four viral elements are not only hyper-targeted, but are also predicted to infect members of different phyla (phylum Bacteroidota and phylum Firmicutes). Members of these two phyla (LW2_63_Bacteroidales, and LW2_3_ Acholeplasmataceae_A) also shared a viral element in the 2016 network (v80_2016, Supplemental Figure 3), but only LW2_3_ Acholeplasmataceae_A hyper-targeted the viral element in that case.

We posit that the shared hyper-targeting across multiple phyla is a result of LGT or misbinning of CRISPR-encoding scaffolds. CLC_107_Bacteroidales_2017 scaffold Ga0265294_10000314 (length of 63,905 bp, 147 spacers encoded) has a six mismatch, 4,167 bp alignment with

LW2_3_Acholeplasmataceae_A_2016 scaffold Ga0172382_10051677 (length of 4,167bp, 63 spacers encoded) and thus mutual hyper-targeting between distinct phyla is likely due to a misbin of scaffold Ga0172382_10051677. Furthermore, LW2_114_Bacteroidales_2017 scaffold Ga0265293_10000924 (length 67,619 bp, 182 spacers encoded) has a single mismatch 3,322bp alignment with LW2_22_Acholeplasmataceae_2016 scaffold Ga0172382_10071763 (length 3,322bp, 34 spacers encoded) and thus mutual hyper-targeting between distinct phyla is likely due to a misbin of scaffold Ga0172382_10071763. LW2_22_Acholeplasmataceae_2016 and LW2_114_Bacteroidales_2017 also each encode two other CRISPR arrays that share five and two spacers, respectively, between MAGs.

Despite the striking differences between their viral element targeting profiles, CLC_107_Bacteroidales_2017 and LW2_114_Bacteroidales_2017 are predicted to have an Average Nucleotide Identity (ANI) of >98.5 according to dRep[6]. It is noteworthy to mention that despite their high genetic identity, CLC_107_Bacteroidales_2017 and LW2_114_Bacteroidales_2017 do not share a single spacer between any of their CRISPR arrays, however, CLC_107_Bacteroidales_2017 shares six spacers with LW2_22_Acholeplasmatceae_2016. Lastly, LW2_3_Acholeplasmataceae_A_2016 shares one spacer with LW2_114_Bacteroidales_2017 and five spacers with LW2_22_Acholeplasmataceae_2016.

Virally-encoded CRISPR arrays convolute virus-host networks

While linking hosts to their putative viruses using CRISPR spacer matches to protospacers, we also came across instances of viral elements that encoded CRISPR arrays and CRISPR-Cas systems. Virus-encoded CRISPR arrays and CRISPR-Cas systems have been identified before [5,7–14] and have been shown to be involved in crippling host viral defense systems[7] and are suggested

to play roles in regulating host transcription and translation[11], CRISPR-Cas system inhibition[5] and interviral conflicts[5,8]. In the case of interviral conflicts, a CRISPR-encoding virus, integrated as a provirus, can provide its host with immunity against other viruses, akin to a superinfection exclusion system[5]. For this work, we used the full set of viral predictions prior to clustering with CD-HIT[15] in order to preserve CRISPR array variation between closely-related viral elements.

Spacers encoded in viral CRISPR arrays from 2016 were predicted to target 89 viral elements in the unclustered 2016 virus data set. Omitting spacer matches between viral CRISPR arrays, spacers encoded in 2016 virus CRISPR arrays were predicted to target 32 viral elements in the unclustered 2016 virus dataset.

We next wanted to assess if any of the viral elements targeted by viral CRISPR arrays were also targeted by host MAGs in any of our networks. Wherever this was the case, we wanted to determine if the CRISPR immunity against the viral element came from a CRISPR-encoding provirus within the host MAG. For this, we screened relevant host MAGs for proviruses using a homology-based approach[16] (see methods for details), followed by Prophage Hunter[17], which in select cases was also followed by PHASTER[18]. CRISPR arrays in putative provirus elements were annotated using CRISPRCasFinder[19].

In the 2016 network, only one viral element (v30_2016) shown previously to be targeted by viral CRISPR arrays, was targeted by a host MAG (CLC_T2_12_Fibrobacteraceae). Notably, v30_2016 itself was one of the viral elements predicted to encode its own CRISPR array. In the case of CLC_T2_12_Fibrobacteraceae, no prophages were detected. In the 2017 host to 2016 virus network, 11 of 89 viral elements targeted by viral CRISPR arrays were implicated in the network, targeted by a total of 10 unique hosts. Screening first for proviruses using the homology-based approach predicted six provirus-containing host genomes. Prophage hunter agreed with 4/6 of

these predictions. 2/4 of these provirus regions had high confidence CRISPR arrays annotated within them by CRISPRCasFinder.

LW1_9_Paceibacterales (completion: 73.56%, contamination: 5.17%) encodes a putative prophage of v33_2016, a virus that contains a CRISPR array that targets its free virus equivalent via six spacers in our 2017-host-to-2016-virus network. LW1_9_Paceibacterales also contains a second CRISPR array, not within a prophage, that targets v33_2016 two additional times. Second, LW3_16_Pacearchaeales (completion: 71.88%, contamination: 0.00%) encodes a provirus of v169_2016. Notably, the scaffold that contains the putative provirus, Ga0378408_0001874, is predicted to encode five CRISPRs and a total of 40 spacers according to CRISPRCasFinder, all of which are encoded within the predicted proviral region. This host's provirus also targets its free virus equivalent, v169_2016, in the 2017 host to 2016 virus network 27 times, which stem from the provirus-encoded array. Unlike in the case of LW1_9_Paceibacterales, LW3_16_Pacearchaeales does not encode CRISPR immunity against v169_2016 outside of its provirus. Lastly, LW2_146_Pigmentiphaga (completion: 97.56%, contamination: 0.30%), contains a prophage of v36_2016 which encodes two Cas gene clusters according to CRISPRCasFinder: a Cas-TypeIV cluster containing *Csf2_1_IV*, and *Csf3_1_IV*, and a Cas-TypeIA cluster containing *Cas6_0_IA*. In this same host, ~37Kbp upstream of the Cas gene clusters, but outside the prophage boundary predicted by Prophage Hunter, a mini-CRISPR array[8] (containing two spacers according to CRISPR-CasFinder, three spacers according to the JGI's standard operating pipeline) was annotated. When the free viral element, v36_2016 (predicted as circular by VirSorter) was put through CRISPR-Cas Finder, it was predicted to contain the same Cas gene clusters and CRISPR array as LW2_146_Pigmentiphaga's putative prophage-containing scaffold. This could suggest that the prophage introduced CRISPR-Cas systems into its host's

genome upon integration or that the CRISPR-Cas systems of this host were co-opted by its virus after excision from the host chromosome. The CRISPR array being outside the prophage boundary predicted by Prophage Hunter is likely due to partial degradation of the prophage over time. LW2_146_ Pigmentiphaga has three connections to v36_2016 in the 2017-host-to-2016-virus network, all of which are predicted to be due to prophage-mediated immunity.

We next repeated the workflow described above to examine CRISPR arrays in the viral elements identified in our 2017 data - 91 (0.48%) were predicted to encode CRISPR arrays. Unlike what was observed with our 2016 viral elements, here there were 8 instances of shared CRISPR spacers between CRISPR-encoding viral elements.

Spacers encoded in 2017 viral CRISPR arrays were predicted to target 117 viral elements in the unclustered 2017 virus data set. Accounting for matches between viral CRISPR arrays, spacers encoded in 2017 virus CRISPR arrays were predicted to target 44 viral elements in the unclustered 2017 dataset. Four viral elements target by viral CRISPR arrays are implicated in our 2016-hosts-to-2017 viruses network and are targeted by a total of three hosts. None of these three host MAGs was predicted to contain a CRISPR-encoding provirus.

In our 2017 network, 10 viral elements targeted by viral CRISPR arrays were within the network and were targeted by a total of 9 hosts. Only one of the hosts targeting any of these 11 viral elements was predicted to contain a prophage: LW1_30_Synergistaceae. Homology-based methods and PHASTER, but not Prophage Hunter, identified putative prophage elements from a single scaffold in LW1_30_ Synergistaceae. Though CRISPRCasFinder did not identify a CRISPR in these putative prophage regions, manual inspection of the JGI CRISPR annotations revealed a CRISPR within the scaffold from positions 658:788. The two spacers in this region are also responsible for the targeting of three viral elements, v159_2017, v91_2017, and v60_2017, by

LW1_30_Synergistaceae in our 2017 host to 2017 virus network. Notably, v159_2017 is predicted to be the same as the viral element integrated within the MAG as a prophage. This CRISPR array, however, only had partial overlap with the 779:10068 prophage region predicted by PHASTER. Since provirus boundary prediction methods are still very limited in their accuracy, we conservatively refute that a CRISPR-encoding prophage is within the LW1_30 scaffold.

In summary, for the 2016 viruses in the 2016 network, no provirus-mediated interviral conflicts were identified. In the 2017-host-to-2016-virus network however, while no genuine interviral conflicts were identified, shared CRISPR spacers between proviruses and their free virus equivalents convoluted our virus-host networks. In the case of the 2017 viruses, both in the 2016-host-to-2017-virus network and 2017-host-to-2017-virus networks, no provirus-mediated interviral conflicts were found. We have shown that the presence of CRISPR-encoding viral elements can convolute virus-host networks: if a MAG contains a provirus that encodes a CRISPR array and the non-integrated equivalent of said provirus is also present in the metagenomic assemblies, then provirus CRISPR spacer to free virus CRISPR spacer matches could be what draws a host-virus connection. This could be further confounded by two distinct viral elements or other mobile genetic elements sharing a spacer between their CRISPR arrays.

References

1. Huntemann, M. *et al.* The standard operating procedure of the DOE-JGI Microbial Genome Annotation Pipeline (MGAP v.4). *Stand. Genom. Sci.* **10**, 86 (2015).

2. von Meijenfeldt, F. A. B., Arkhipova, K., Cambuy, D. D., Coutinho, F. H. & Dutilh, B. E. Robust taxonomic classification of uncharted microbial sequences and bins with CAT and BAT. *Genome Biol.* **20**, 217 (2019).
3. Chaumeil, P.-A., Mussig, A. J., Hugenholtz, P. & Parks, D. H. GTDB-Tk: a toolkit to classify genomes with the Genome Taxonomy Database. *Bioinformatics* **36**, 1925–1927 (2019).
4. Makarova, K. S. *et al.* Evolutionary classification of CRISPR–Cas systems: a burst of class 2 and derived variants. *Nat. Rev. Microbiol.* **18**, 67–83 (2020).
5. Faure, G. *et al.* CRISPR–Cas in mobile genetic elements: counter-defence and beyond. *Nat. Rev. Microbiol.* **17**, 513–525 (2019).
6. Olm, M. R., Brown, C. T., Brooks, B. & Banfield, J. F. dRep: a tool for fast and accurate genomic comparisons that enables improved genome recovery from metagenomes through de-replication. *ISME J.* **11**, 2864–2868 (2017).
7. Seed, K. D., Lazinski, D. W., Calderwood, S. B. & Camilli, A. A bacteriophage encodes its own CRISPR/Cas adaptive response to evade host innate immunity. *Nature* **494**, 489–491 (2013).
8. Medvedeva, S. *et al.* Virus-borne mini-CRISPR arrays are involved in interviral conflicts. *Nat. Commun.* **10**, 5204 (2019).
9. Minot, S. *et al.* The human gut virome: Inter-individual variation and dynamic response to diet. *Genome Res.* **21**, 1616–1625 (2011).
10. Minot, S. *et al.* Rapid evolution of the human gut virome. *PNAS* **110**, 12450–12455 (2013).
11. Al-Shayeb, B. *et al.* Clades of huge phages from across Earth's ecosystems. *Nature* **578**, 425–431 (2020).

12. Chénard, C., Wirth, J. F. & Suttle, C. A. Viruses Infecting a Freshwater Filamentous Cyanobacterium (*Nostoc* sp.) Encode a Functional CRISPR Array and a Proteobacterial DNA Polymerase B. *mBio* **7**, (2016).

13. Hargreaves, K. R., Flores, C. O., Lawley, T. D. & Clokie, M. R. J. Abundant and Diverse Clustered Regularly Interspaced Short Palindromic Repeat Spacers in Clostridium difficile Strains and Prophages Target Multiple Phage Types within This Pathogen. *mBio* **5**, (2014).

14. Al-Shayeb, B. *et al.* Diverse virus-encoded CRISPR-Cas systems include streamlined genome editors. *Cell* **185**, 4574-4586.e16 (2022).

15. Li, W. & Godzik, A. Cd-hit: a fast program for clustering and comparing large sets of protein or nucleotide sequences. *Bioinformatics* **22**, 1658–1659 (2006).

16. Emerson, J. B. *et al.* Host-linked soil viral ecology along a permafrost thaw gradient. *Nat. Microbiol.* **3**, 870–880 (2018).

17. Song, W. *et al.* Prophage Hunter: an integrative hunting tool for active prophages. *Nucleic Acids Res.* **47**, W74–W80 (2019).

18. Arndt, D. *et al.* PHASTER: a better, faster version of the PHAST phage search tool. *Nucleic Acids Res.* **44**, W16–W21 (2016).

19. Couvin, D. *et al.* CRISPRCasFinder, an update of CRISRFinder, includes a portable version, enhanced performance and integrates search for Cas proteins. *Nucleic Acids Res.* **46**, W246–W251 (2018).

20. Dion, M., Labrie, S., Shah, S. & Moineau, S. CRISPRStudio: A User-Friendly Software for Rapid CRISPR Array Visualization. *Viruses* **10**, 602 (2018).

21. Kearse, M. *et al.* Geneious Basic: An integrated and extendable desktop software platform for the organization and analysis of sequence data. *Bioinformatics* **28**, 1647–1649 (2012).

Supplemental Tables and Figures

Supplemental Table 1: Putative cross-phylum and cross-domain interaction based on host MAG CRISPR spacers and viral element protospacer connections, with categories for curation listed. Grey shading indicates categories where a given interaction failed curation.

Putative hosts	Host MAG phylum (GTDB-tk)	Host CRISPR scaffold phylum (JGI, Contig Annotation Tool)	Host completion & contamination (%)	Host CRISPR scaffold lengths (bp)	# shared CRISPR spacers	Predicted viral element(s)	Overall
**Cross-Phylum Infection Predictions**							
LW2_138_Bacilli_2017<br>LW2_137_Muirbacterieaceae_2017<br>LW2_139_Absconditabacteriales_2017	Firmicutes<br>Muirbacteria<br>Patescibacteria	Proteobacteria, Muirbacteria<br>Firmicutes, Muirbacteria<br>Planctomycetes, Bacteria	72.12, 5.33<br>93.26, 4.56<br>70.53, 3.61	5,475<br>50,129<br>16,695	0	v2_2017<br>v154_2016	pass
LW2_36_Ottowia_2017<br>GW1_223_Dojkabacteria_2017	Proteobacteria<br>Patescibacteria	Unknown<br>Viral/mixed bacteria/Euryarchaeota, cellular organisms/no rank	95.56, 3.26<br>80.02, 4.08	7,569<br>78,174	0	v102_2017	pass
LW2_146_Pigmentiphaga_2017<br>LW2_50_Polyangia_2017	Proteobacteria<br>Myxococcota	Proteobacteria, Bacteria<br>Proteobacteria	97.56, 0.30<br>91.20, 4.52	89,231<br>87,942	0	v36_2016	pass
GW1_158_Paceibacteria_2017<br>LW4_67_Methylobacteria_2017	Patescibacteria<br>Proteobacteria	Unknown, Candidatus Riflebacteria<br>Proteobacteria	82.89, 1.88<br>97.62, 3.88	4,111<br>15,464	56	v42_2016	pass
LW2_63_Bacteroidales_2016<br>LW2_22_Acholeplasmataceae_2016<br>LW2_3_Acholeplasmataceae_A_2016	Bacteroidota<br>Firmicutes<br>Firmicutes	Bacteroidetes, no rank<br>Bacteroidetes,<br>Bacteroidetes/Chlorobi<br>Unknown	91.94, 0.90<br>94.48, 3.01<br>73.36, 0.69	21,376<br>3,322<br>4,167	0	v80_2016	fail
LW2_30_Dehalococcoida_2017<br>LW2_58_Limnochordia_2017	Chloroflexota<br>Firmicutes	Proteobacteria, Bacteria<br>Bacteroidetes/Proteobacteria, cellular organisms/no rank	86.44, 5.02<br>82.80, 9.32	4,005<br>5,396	0	v35_2017<br>v123_2017	fail
**Cross-Domain Infection Predictions**							
CLC_82_Cloacimonadaceae_2017<br>CLC_33_Methanoculleus_2017	Cloacimonadota<br>Halobacterota	Mixed bacteria/Euryarchaeota,<br>Cloacimonetes<br>Unknown	95.05, 3.09<br>96.62, 3.27	6,990<br>3,667	0	v165_2016	fail
LW3_15_Cloacimonadaceae_2017<br>CLC_33_Methanoculleus_2017	Cloacimonadota<br>Halobacterota	Cloacimonetes<br>Unknown	97.74, 6.19<br>96.62, 3.27	40,641<br>3,667	0	v178_2017<br>v147_2017	fail
CLC_92_Sphaerochaeata_2017<br>CLC_33_Methanoculleus_2017	Spirochaetota<br>Halobacterota	Unknown<br>Unknown	87.04, 4.21<br>96.62, 3.27	3,829<br>3,710	0	v172_2017<br>v181_2017	fail
CLC_110_Methanoculleus_2017<br>CLC_67_Massilibacteroides_2017	Halobacterota<br>Bacteroidota	Euryarchaeota<br>Euryarchaeota	76.43, 9.31<br>70.44, 9.71	15,438<br>10,142	0	v39_2017	fail

Supplemental Tables S2-S7 are available as a single .xlsx file for download online.

Supplemental Table S2: Host MAGs and predicted viral elements included in the 2016 host to 2016 viruses network, including number of spacer matches and coverages for both partners.

Supplemental Table S3: Host MAGs and predicted viral elements included in the 2017 host to 2017 viruses network, including number of spacer matches and coverages for both partners.

Supplemental Table S4: Host MAGs and predicted viral elements included in the 2016 host to 2017 viruses network, including number of spacer matches and coverages for both partners.

Supplemental Table S5: Host MAGs and predicted viral elements included in the 2017 host to 2016 viruses network, including number of spacer matches and coverages for both partners.

Supplemental Table S6: Networked MAGs and their predicted taxonomy, genome statistics, and NCBI accessions.

Supplemental Table S7: Connection between VirSorter scaffold ID, simplified name used in article text, and IMG accession for key predicted viral elements.

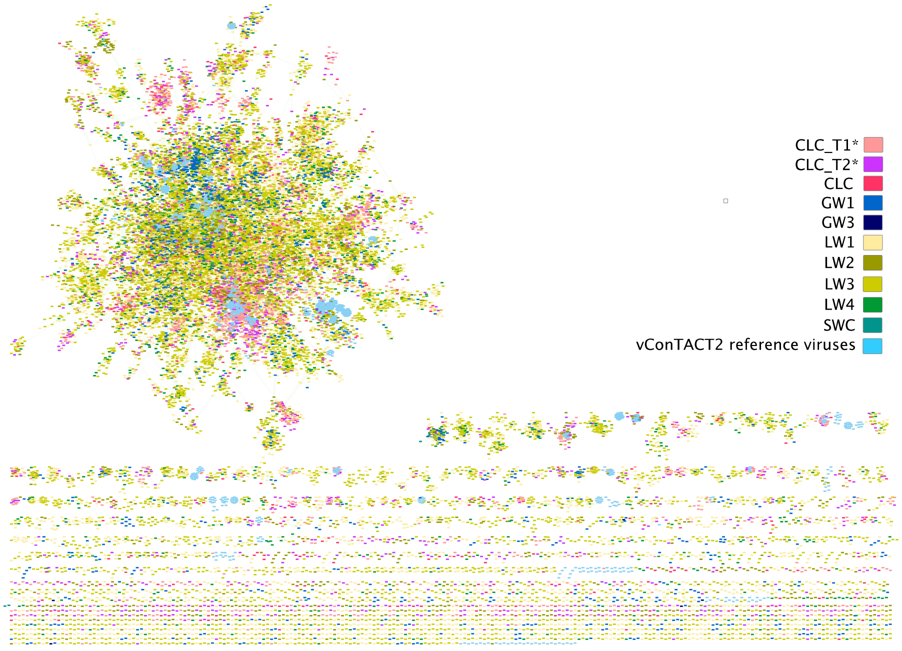

Supplemental Figure 1: Viral diversity across distinct samples. vConTACT2 was used to generate gene-sharing networks for all 2016 and 2017 viral elements. Edges connecting nodes are not shown. CLC samples have non-overlapping colors to distinguish the two 2016 time-points.

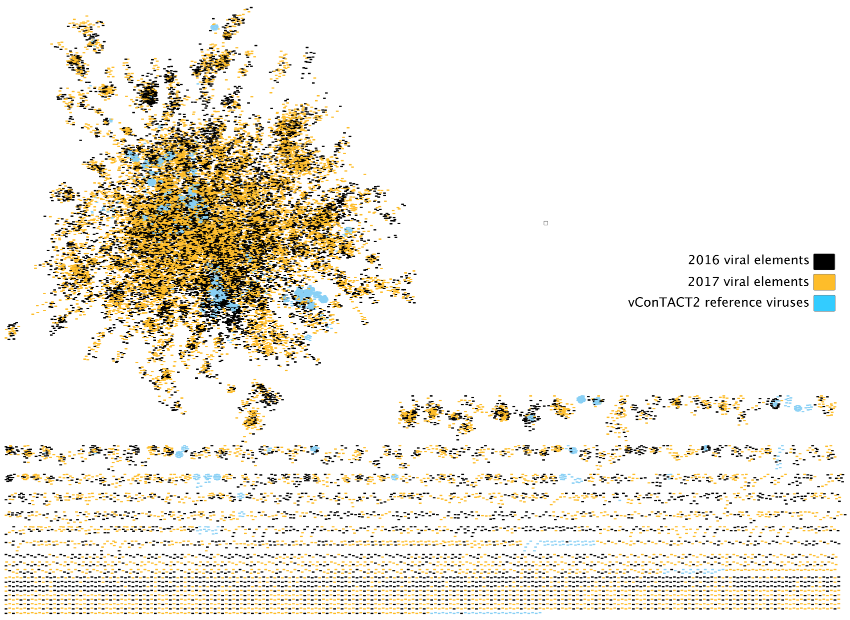

Supplemental Figure 2: Viral diversity across distinct timepoints. vConTACT2 was used to generate gene-sharing networks for all 2016 and 2017 viral elements. Edges connecting nodes are not shown.

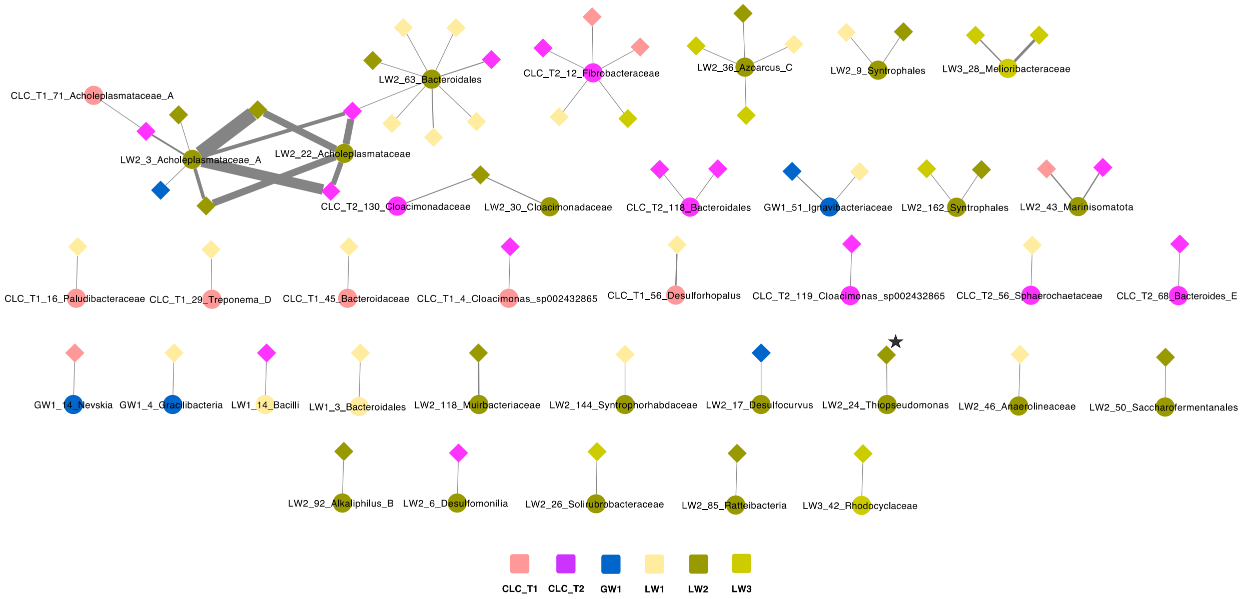

Supplemental Figure 3: Virus-host interactions in a Southern Ontario landfill (2016) with host MAG taxonomy. CRISPR array spacers from the MAGs (circles) were mapped against the viral elements (diamonds) predicted by VirSorter and VIBRANT to establish virus-host linkages (connecting edges). The network is colored by the geographic sampling location from which the MAG or viral element originated, with the width of the edges proportional to the number of spacer-protospacer matches supporting the connection. MAG identifier and lowest level of taxonomy as determined by GTDB-Tk are noted for networked MAGs. A viral element encoding a Diversity Generating Retroelement (DGR) is denoted by a star.

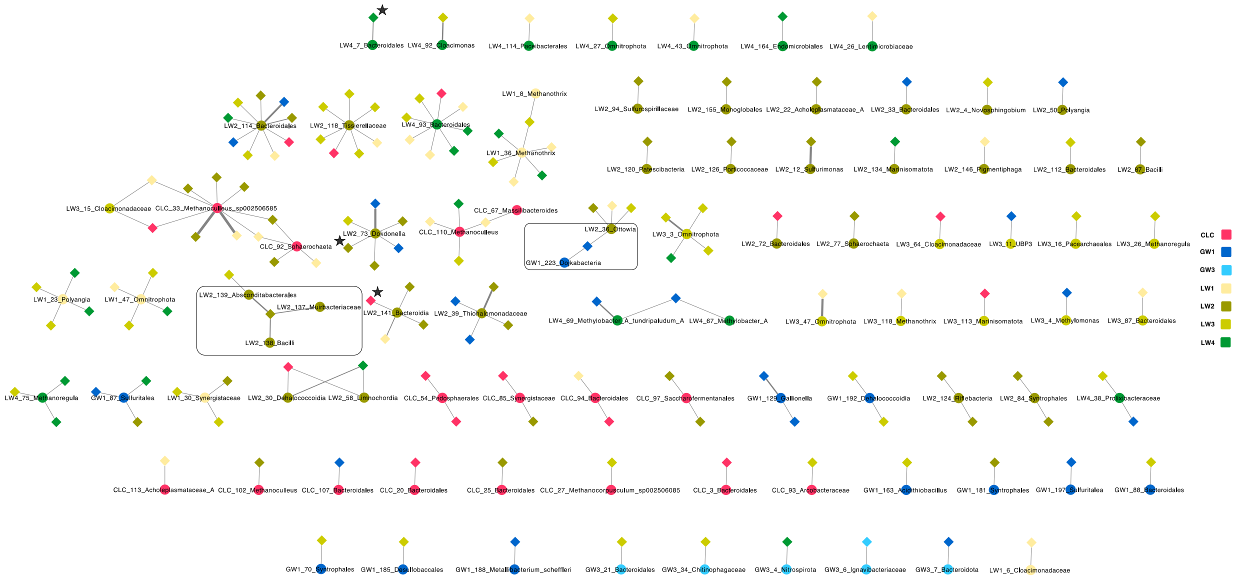

Supplemental Figure 4: Virus-host interactions in a Southern Ontario landfill (2017). CRISPR array spacers from the MAGs (circles) were mapped against the viral elements (diamonds) predicted by VirSorter and VIBRANT to establish virus-host linkages (connecting edges). The network is colored by the geographic sampling location from which the MAG or viral element originated, with the width of the edges proportional to the number of spacer-protospacer matches supporting the connection. MAG identifier and lowest level of taxonomy as determined by GTDB-Tk are noted for networked MAGs. Three viral elements encoding Diversity Generating Retroelements (DGRs) are denoted by stars. Viral elements and hosts involved in putative cross-phylum connections are highlighted with boxes.

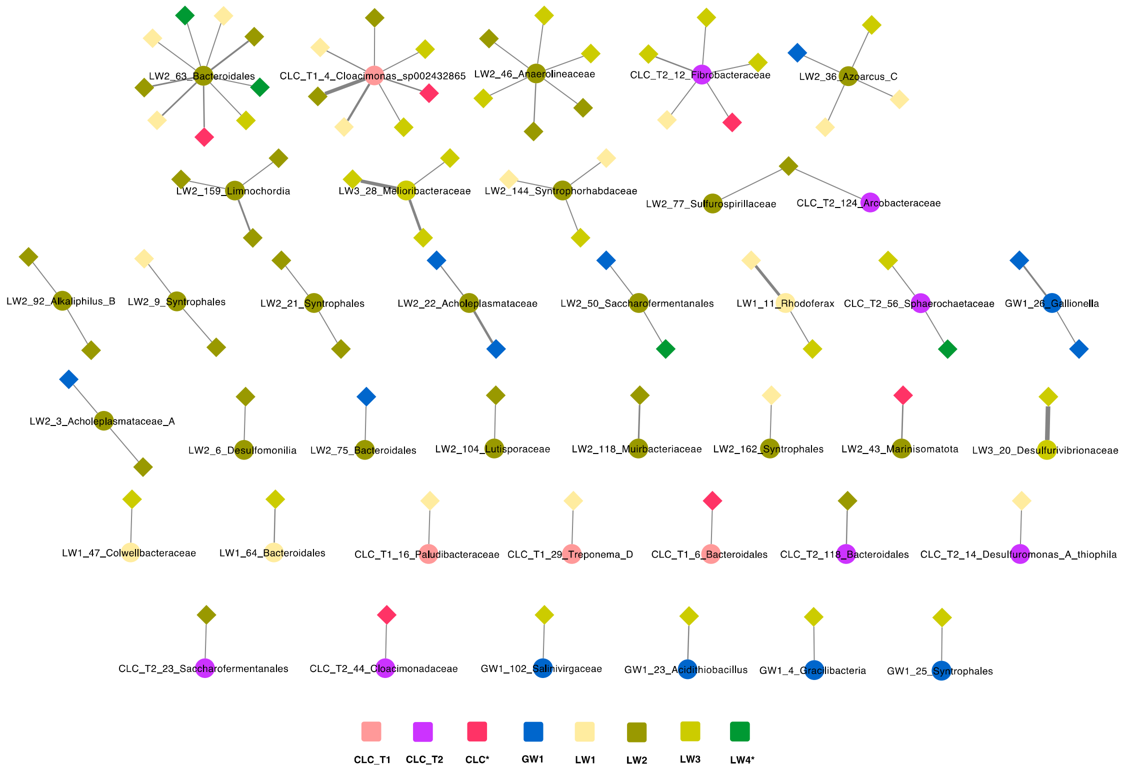

Supplemental Figure 5: Virus-host interactions in a Southern Ontario landfill (2016 hosts to 2017 viruses). CRISPR array spacers from the MAGs (circles) were mapped against the viral elements (diamonds) predicted by VirSorter and VIBRANT to establish virus-host linkages (connecting edges). The network is colored by the geographic sampling location from which the MAG or viral element originated, with the width of the edges proportional to the number of spacer-protospacer matches supporting the connection. MAG identifier and lowest level of taxonomy as determined by GTDB-Tk are noted for networked MAGs.

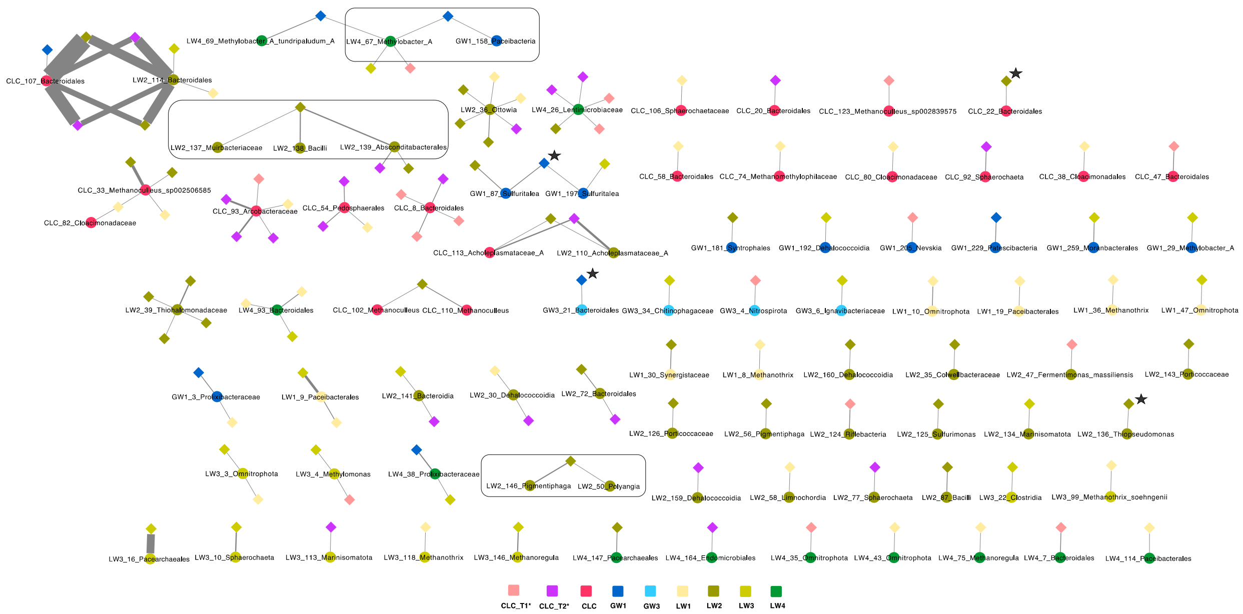

Supplemental Figure 6: Virus-host interactions in a Southern Ontario landfill (2017 hosts to 2016 viruses). CRISPR array spacers from the MAGs (circles) were mapped against the viral elements (diamonds) predicted by VirSorter and VIBRANT to establish virus-host linkages (connecting edges). The network is colored by the geographic sampling location from which the MAG or viral element originated, with the width of the edges proportional to the number of spacer-protospacer matches supporting the connection. MAG identifier and lowest level of taxonomy as determined by GTDB-Tk are noted for networked MAGs. CLC samples have non-overlapping colors to distinguish the two 2016 time-points. Four viral elements encoding Diversity Generating Elements (DGRs) are denoted by stars. All viral elements and hosts involved in putative cross-phylum connections are within boxes.

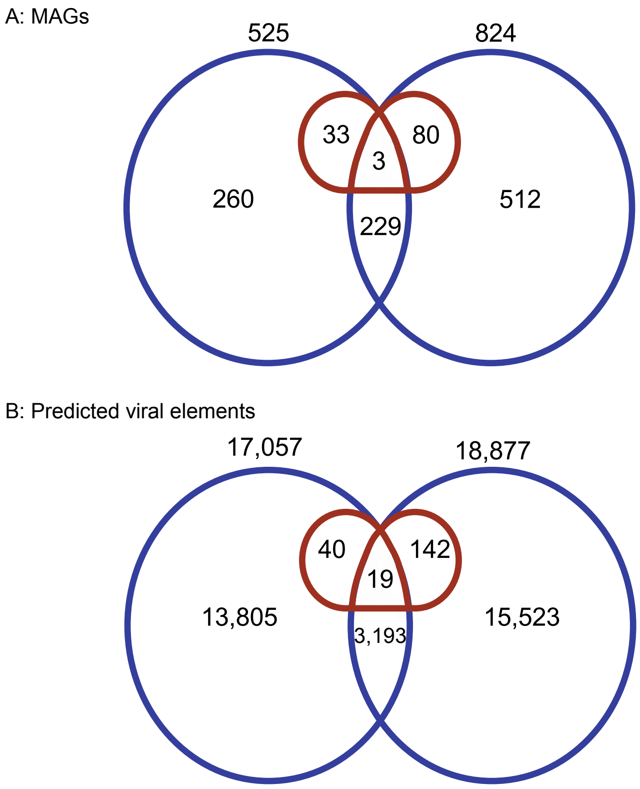

Supplemental Figure 7: Venn diagrams of A: all reconstructed MAGs from the 2016 (left) and 2017 (right) datasets, with dataset overlaps between all MAGs (blue) and networked MAGs (brown) indicated by the overlapping central region. B: all predicted viral elements from the 2016 (left) and 2017 (right) datasets, with dataset overlaps between all viral elements (blue) and networked viral elements (brown) indicated by the overlapping central region.

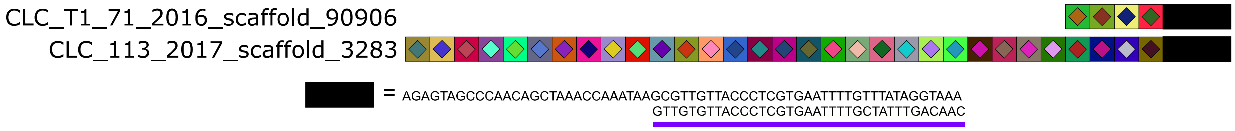

Supplemental Figure 8: CRISPR element targeting the same viral element between 2016 and 2017 showing changes in CRISPR array spacers (colored blocks) and one conserved flanking sequence (black rectangle). The direct repeat consensus sequence shared between the two arrays is shown under the flanking sequence and is underlined in purple. The similarity between a subsequence of the flanking sequence and the direct repeat consensus suggests that the flanking sequence may contain a partially-degraded repeat along with a 28 bp spacer upstream of it. Omitting the possible spacer within the flanking sequence, none of the spacers between the two arrays are shared despite near-identical host genomes from the CLC in 2016 and 2017. The backbone of this figure was generated through the use of CRISPRStudio[20].

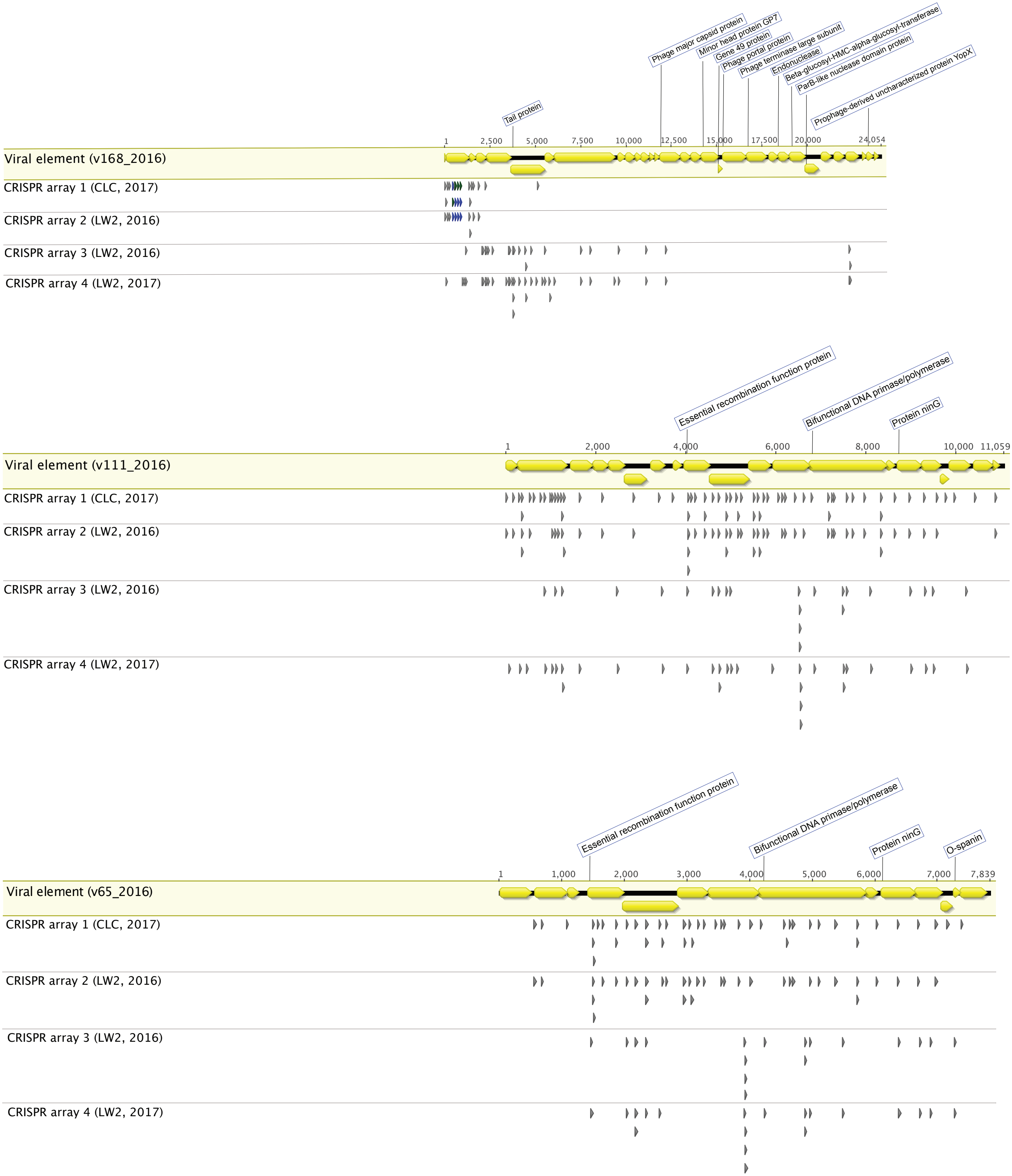
Phage major capsid protein
Minor head protein GP7
Gene 49 protein
Phage portal protein
Phage terminase large subunit
Endonuclease
Beta-glucosyl-HMC-alpha-glucosyl-transferase
ParB-like nuclease domain protein
Prophage-derived uncharacterized protein YopX
Tail protein
1
2,500
5,000
7,500
10,000
12,500
15,000
17,500
20,000
24,054
Viral element (v168_2016)
CRISPR array 1 (CLC, 2017)
CRISPR array 2 (LW2, 2016)
CRISPR array 3 (LW2, 2016)
CRISPR array 4 (LW2, 2017)
Essential recombination function protein
Bifunctional DNA primase/polymerase
Protein ninG
1
2,000
4,000
6,000
8,000
10,000
11,059
Viral element (v111_2016)
CRISPR array 1 (CLC, 2017)
CRISPR array 2 (LW2, 2016)
CRISPR array 3 (LW2, 2016)
CRISPR array 4 (LW2, 2017)
Essential recombination function protein
Bifunctional DNA primase/polymerase
Protein ninG
O-spanin
1
1,000
2,000
3,000
4,000
5,000
6,000
7,000
7,839
Viral element (v65_2016)
CRISPR array 1 (CLC, 2017)
CRISPR array 2 (LW2, 2016)
CRISPR array 3 (LW2, 2016)
CRISPR array 4 (LW2, 2017)

Supplemental Figure 9: Genetic features targeted by host CRISPR arrays on hyper-targeted viral elements. The coding sequences' annotations for the hyper-targeted viral elements are displayed. CRISPR spacers were mapped to regions of the viral element, with the mapping and visualization performed in Geneious[21]. In the targeting profile of a single host, a non-grey triangle represents a single spacer that had multiple matches to regions of the viral element. When shared, these single spacers are coloured the same between distinct host targeting profiles.
